# Systematic review and meta-analysis of early visual processing, social cognition, and functional outcomes in schizophrenia spectrum disorders^☆^

**DOI:** 10.1016/j.scog.2025.100351

**Published:** 2025-02-15

**Authors:** Akke Ganse-Dumrath, Anya Chohan, Steven Samuel, Paul Bretherton, Corinna Haenschel, Anne-Kathrin Fett

**Affiliations:** a Department of Psychology, School of Health and Medical Sciences, City St George's, University of London, UK; b Department of Psychosis Studies, Institute of Psychiatry, Psychology, and Neuroscience, King's College London, UK

**Keywords:** Non-affective psychosis, Psychosocial functioning, Perceptual abnormalities, Social cognitive deficits, Mediation

## Abstract

Non-affective psychotic disorders are marked by cognitive and sensory processing abnormalities, including in early visual processing and social cognition. Understanding the relationships between these deficits and their impact on daily-life functional outcomes may help to improve outcomes in affected individuals. This systematic review and meta-analysis aimed to summarise the existing evidence on the relationships between early visual processing, social cognition, and functional outcomes, and to assess the evidence regarding the mediating role of social cognition in the association between early visual processing and functional outcomes in individuals with schizophrenia spectrum disorders. A comprehensive search across five databases identified 364 potentially eligible studies, with eight articles meeting all inclusion criteria. Meta-analytic techniques were employed to synthesise effect sizes and assess a meta-mediation model. Three random-effects meta-analyses revealed significant associations between all three domains of interest. Social cognition partially mediated the relationship between early visual processing and functional outcomes. The direct effect of early visual processing on functional outcomes remained significant, albeit with a reduced effect size. The findings suggest that interventions targeting both early visual processing and social cognition concurrently may improve functional outcomes more effectively than focusing on either domain alone.

## Highlights

• Aberrant early visual processing is related to poorer functional outcomes.
• Aberrant early visual processing is related to social cognition impairments.
• Social cognition mediates the relationship between early visual processing and functional outcomes.
• Targeting early visual processing and social cognition concurrently may improve functional outcomes.

## 1 Introduction

Non-affective psychotic disorders are characterised by a spectrum of symptoms that extend beyond the commonly known psychotic manifestations, such as delusions and hallucinations. For instance, individuals with schizophrenia spectrum disorders (SSDs)^2^ experience a wide range of cognitive and perceptual abnormalities that are related to functional outcomes (FOs) (Haenschel et al., 2007; Kim et al., 2005; Li et al., 2020). FOs encompass various aspects of daily functioning, including work productivity, independent living skills, self-care abilities, and the quality of immediate and extended social relationships, often referred to as social functioning (Alkan et al., 2021; Bowie and Harvey, 2006; Tandon et al., 2009).

Research in the field has traditionally focused on higher-order cognitive functions, such as memory, attention, and executive functions, which have consistently been found to be impaired in SSDs, as evidenced by large meta-analyses (Catalan et al., 2024; Heinrichs and Zakzanis, 1998). Studies have reported robust moderate effect size associations between most non-social cognitive domains and different FOs (Fett et al., 2011; Green et al., 2005a; Halverson et al., 2019). The Measurement and Treatment Research to Improve Cognition in Schizophrenia (MATRICS) initiative has further highlighted the potential role of social cognition (SC) in influencing FOs in individuals with SSDs (Buchanan et al., 2005; Nuechterlein et al., 2004). SC refers to cognitive skills required for perceiving, interpreting, and responding to social information, with key domains including social perception and knowledge, Theory of Mind (ToM), and emotion perception and processing (Green et al., 2005a). These skills are essential for effective social interactions and are often compromised in individuals with SSDs (Kohler et al., 2010; Langdon et al., 2002; Savla et al., 2013; Sprong et al., 2007). Consequently, SC deficits are thought to contribute to social and occupational dysfunction by impairing the ability to interpret social cues, understand others' intentions, and regulate emotions (Frith, 2008; Green et al., 2015). Two large meta-analyses suggest that SC explains additional variance in FOs beyond non-social cognition, though variability exists across SC domains, such as ToM and emotion perception (Fett et al., 2011; Halverson et al., 2019).

Beyond higher-order cognitive processes, there has also been a continued interest in more fundamental sensory processes, particularly early visual processing (EVP) and its potential impact on SC and FOs (Haenschel et al., 2007; Diamond et al., 2022; Herrmann et al., 2004; Javitt, 2009a; Sergi and Green, 2003). EVP refers to the initial stages of visual information processing, where basic perceptual features such as colour, brightness, shape, and contrast are decoded (Butler et al., 2005; McCleery et al., 2020). Key sub-processes of EVP include contrast sensitivity (detecting differences between light and dark), luminance detection (perceiving variations in brightness), contour integration (organising visual contours into coherent shapes), and visual masking (where one visual stimulus affects the perception of another) (Butler et al., 2005; Green et al., 2012a; Silverstein and Keane, 2011). Research has consistently shown that EVP impairments are highly prevalent in SSDs (Butler et al., 2008; Keane et al., 2016). For instance, affected individuals have been found to require higher thresholds to detect light and discern contrast differences compared to healthy controls (Herrera et al., 2021; Yang et al., 2013). Additionally, there is evidence that individuals with SSDs exhibit difficulties in integrating visual elements into a coherent whole and have a limited capacity for perceiving multiple visual details simultaneously (Keane et al., 2014; Silverstein and Rosen, 2015).

Given the importance of visual input in navigating social interactions, EVP deficits have been linked to both SC impairments and poorer FOs (McCleery et al., 2020; Green et al., 2012a; Herrera et al., 2021; Brittain et al., 2010; Rassovsky et al., 2011; Sergi et al., 2006). Some studies propose that SC may act as a mediator between EVP and FOs, whereby difficulties in decoding basic visual information cascade into impairments in processing socially relevant stimuli, ultimately contributing to functional impairments (Green et al., 2012a; Brittain et al., 2010; Rassovsky et al., 2011; Sergi et al., 2006). However, direct evidence supporting this mediation model remains limited, with most studies being cross-sectional and only a few explicitly testing mediation.

One explanation for the potential EVP → SC → FO pathway is that EVP deficits may consume significant cognitive resources, reducing the capacity for higher-order tasks such as interpreting social cues or engaging in ToM (Green et al., 2012a). Additionally, EVP deficits may introduce noise into the perceptual system, distorting subsequent higher-order cognitive processes. Disruptions in early visual pathways - such as the retina, optic nerve, and thalamus - can lead to abnormal sensory integration, reducing the accuracy of conscious perception (Butler et al., 2007; Javitt and Freedman, 2015; Silverstein et al., 2015). A decreased signal-to-noise ratio in these pathways may result in a reliance on incomplete or incorrect visual input, affecting downstream decision-making processes and leading to compensatory mechanisms such as excessive amplification of sensory and noise signals or inhibition of pre-processing due to increased errors (de Lecea et al., 2012; Silverstein et al., 2020). This instability in bottom-up visual signals may further prevent the formation of stable internal models, affecting predictive coding and contextual modulation (Adámek et al., 2022). Consequently, lower-level sensory processes are increasingly recognised as potential bottlenecks that may not only consume cognitive resources but also impair the accuracy of sensory representations, restricting higher-order cognitive functioning and subsequently impacting FOs (Haenschel et al., 2007; Butler et al., 2005; McCleery et al., 2020; Green and Nuechterlein, 1999).

While these findings support an EVP → SC → FO pathway, the relationships between these domains may be more complex. SC impairments, for instance, might also impact EVP by influencing attentional allocation, modifying sensory input processing over time, or altering top-down perceptual expectations (Silverstein et al., 2020). Additionally, broader neurodevelopmental factors or disruptions in global cognitive functioning could underlie both EVP and SC deficits, making it difficult to determine a strict causal hierarchy (Foss-Feig et al., 2017; Javitt, 2023).

To our knowledge, findings in this field have not been systematically summarised. Therefore, this systematic review and meta-analysis aims to provide a quantitative synthesis of the relationships between EVP, SC, and FOs in SSDs. Specifically, we aim to (1) examine the strength of these relationships, (2) assess the mediating role of SC between EVP and FOs, and (3) explore potential moderating effects of demographic and clinical variables on these relationships across the available literature.

## 2 Methods

### 2.1 Protocol registration and reporting standards

This meta-analysis is registered with the International Prospective Register of Systematic Reviews (PROSPERO) under the registration number CRD42023473171. All procedures align with the Preferred Reporting Items for Systematic Reviews and Meta-Analyses (PRISMA) guidelines (Page et al., 2021).

### 2.2 Search strategy

On November 10, 2023, a comprehensive search was conducted across multiple databases, including Academic Search Ultimate, APA PsychInfo, MEDLINE Complete, Embase, and Web of Science. Additionally, reference lists of eligible studies and reviews were examined for undetected references.

The search strategy employed a combination of key terms and controlled vocabulary, such as Medical Subject Heading (MeSH). Search terms related to non-affective psychosis (e.g., schizophrenia, psychosis) and EVP (e.g., early visual processing, visual perception) were combined with terms associated with SC (e.g., emotion processing, ToM) and/or FOs (e.g., vocational functioning, skills of daily living, social functioning).

Filters were applied to limit results to peer-reviewed articles published in English from January 1977 to November 2023. This period was chosen due to the establishment of more standardised criteria and definitions for SSDs, which contributed to greater diagnostic consistency across different diagnostic systems. Detailed search strategies for each database are provided in Supplementary Tables S1-S5.

### 2.3 Eligibility criteria

Eligibility for study inclusion was determined based on several criteria: (1) the sample included individuals diagnosed with non-affective psychosis (i.e., schizophrenia and related disorders) according to established criterion-based diagnostic systems (i.e., International Classification of Diseases or Diagnostic and Statistical Manual of Mental Disorders), (2) participants were aged 16 years or older, and (3) studies provided cross-sectional zero-order bivariate correlations between EVP, SC, and FOs. Studies were excluded if they involved special patient populations (e.g., geriatric patients, patients with childhood psychosis) that could potentially affect cognitive performance differentially.

### 2.4 Study selection

The search yielded 364 potentially eligible articles that were inspected for inclusion. Initial screening of titles and abstracts was conducted by the lead author, with a random 10 % independently screened by a second reviewer (AC). Full texts of potentially relevant studies were then retrieved and independently assessed by the same two reviewers. A decision flowchart (Supplementary Fig. S1) guided the screening process and discrepancies between reviewers were resolved through discussion with the author team. Inter-rater agreement was 92 % for both title/abstract and full-text screenings. Overlapping cohorts were identified by cross-referencing authors and research groups.

### 2.5 Data extraction

Data extraction was conducted by the lead author and cross-verified by another reviewer (AC) using a custom-designed extraction form. Extracted data included publication details (authors, publication year, title, source, study aim(s), and country of origin), study design and methodology (research type, sample size, and study setting), as well as participant demographics and baseline characteristics (age, gender, ethnicity, education, diagnosis, illness duration, medication, and any special characteristics). Moreover, correlation coefficients were extracted. Where multiple tasks were used to assess the same concept, data from all relevant outcome measures were included and averaged. Standardised beta coefficients from one study (Horton and Silverstein, 2008) were transformed into correlation coefficients to ensure consistency with other studies. Any discrepancies between the reviewers during the data extraction process were resolved through discussion with the team.

### 2.6 Outcomes assessed

The outcomes assessed in this meta-analysis were categorised into three key areas. EVP domains included contrast sensitivity, visual masking, span of apprehension, and visual integration. SC domains were categorised according to the NIMH-MATRICS guidelines (Nuechterlein et al., 2004), which grouped tasks into the most common SC domains: Social Perception & Knowledge, ToM, and Emotion Perception & Processing. FOs encompassed work productivity, independent living skills, self-care abilities, and the quality of immediate and extended social relationships. Details of the assessments are provided in Table 1.

**Table 1:** Domains, tests, and parameter descriptions for early visual processing, social cognition, and functional outcomes.

Domain	Test	Parameter description
***Early visual processing***		
1. Contrast Sensitivity (reported by 3 studies)	Freiburg Contrast Test (Bach, 1996)	Thresholds of contrast detection
	Spatial Frequency Gratings (Legge, 1978)	
2. Visual Masking (reported by 4 studies)	Location Masking Task (Green et al., 2002)	Average of the percentage of correct rates
	4-Dot Masking Task (Green et al., 2011)	
3. Span of Apprehension (reported by 1 study)	Partial Report Span of Apprehension Test (Estes and Taylor, 1966)	Reaction time
4. Visual Integration (reported by 1 study)	Jittered Orientation Visual Integration Task (Silverstein et al., 2012)	Accuracy (proportion correct)
***Social cognition***		
1. Social Perception & Knowledge (reported by 4 studies)	Half-Profile of Nonverbal Sensitivity (Ambady et al., 1995)	Percentage of correctly labelled social cues in videotaped scenes
2. Theory of Mind (reported by 2 studies)	Awareness of Social Inference Test (McDonald, 2012)	Number of correctly answered questions
	Hinting Task (Corcoran et al., 1995)	Number of correctly identified hints
3. Emotion Perception & Processing (reported by 2 studies)	Mayer-Salovey-Caruso Emotional Intelligence Test (Mayer et al., 2002)	Number of correctly identified emotions
	Facial Emotion Identification Test (Kerr and Neale, 1993)	Number or percentage of correctly identified emotions in faces
	Facial Emotion Discrimination Test (Kerr and Neale, 1993)	Number of correct distinctions between emotions in faces
***Functional outcome***		
1. Performance-based Measures (reported by 2 studies)	UCSD Performance Based Skills Assessment (Patterson et al., 2001)	Household management, communication, financial skills, transportation, comprehension/planning, medication management
2. Clinician-administered Semi-structured Interviews (reported by 5 studies)	Independent Living Scales Inventory (Loeb, 1996)	Memory/orientation, money management, home and transportation management, health and safety, social adjustment
	Role Functioning Scale (Goodman et al., 1993)	Work productivity, independent living and self-care, immediate and extended social network relationships
3. Informant-report Measures (reported by 1 study)	Multnomah Community Ability Scale (Barker et al., 1994)	Interference with functioning (cognitive and physical factors), adjustment to living in the community, social competence, behavioural problems
4. Self-report Measures (reported by 1 study)	Multidimensional Scale of Independent Functioning (Jaeger et al., 2003)	Independent functioning in work, education, residential domains, role responsibility, presence and level of support, performance quality
	Specific Levels of Functioning Scale (Schneider and Struening, 1983)	Physical functioning, personal care skills, interpersonal relationships, social acceptability, activities of community living, work skills

### 2.7 Study quality assessment

The methodological quality of each study was evaluated using a modified version of the Joanna Briggs Institute (JBI) Critical Appraisal Checklist for Analytical Cross-Sectional Studies (Moola et al., 2024). This checklist includes eight items addressing inclusion criteria, subjects and setting, measurement of condition, exposure, and outcomes, confounding factors, and statistical analysis. Each item on the checklist was rated as ‘yes’ (1 point), ‘no’, or ‘unclear’ (0 points), with total scores reflecting methodological rigour. While all studies were included in the analysis regardless of their methodological quality, the rating was used in the moderator analyses.

### 2.8 Statistical analysis

For each study, we analysed cross-sectional correlations representing: (a) the association between EVP and FOs, (b) EVP and SC, and (c) SC and FOs. In line with previous approaches (Thibaudeau et al., 2023; Ventura et al., 2013), we computed a mean correlation for any study reporting multiple correlations for a given relationship to ensure that each study contributed only once to each relationship. In the majority of studies, positive correlations reflected associations between better EVP or SC performance and better FOs. However, in some cases, higher scores indicated poorer task performance or outcomes, while in others, lower scores did. Accordingly, correlations were recoded so that a positive correlation consistently reflected the association between better cognitive performance and better FOs.

Statistical analyses were performed using Stata (version 18) (StataCorp, 2023) for meta-analyses, and R (version 4.3.2) (R Core Team, 2021) for meta-mediation analysis. Random effects meta-analyses were conducted to estimate effect sizes and 95 % confidence intervals (CIs) for correlation pairs involving three or more studies. Correlations were converted to Fisher's *z* to combine effect sizes and calculate CIs, and then transformed back to *r* values for visual presentation. The strength of correlations was interpreted based on Cohen's conventions: *r* = 0.1 for small effects, *r* = 0.3 for moderate effects, and *r* = 0.5 for large effects (Cohen, 1988).

Between-study heterogeneity was assessed using *I*^2^ and *Q* statistics, following Higgins and Thompson's (2002) guidelines: *I*^2^ = 0.25 for low heterogeneity, *I*^2^ = 0.50 for moderate heterogeneity, and *I*^2^ = 0.75 for substantial heterogeneity. These statistics assess the degree to which the observed effect sizes across studies vary beyond what would be expected due to sampling error alone. Additionally, indices to describe variability in the correlations included *Τ*^2^ (the estimated amount of heterogeneity in the true (transformed) correlations) and *H*^2^ (the total variability in the observed (transformed) correlation coefficients/within-study variance due to sampling error). Publication bias was evaluated with funnel plots and Egger's regression tests.

Subgroup analyses were conducted to compare effect sizes between visual masking tasks (*n* = 3 studies) and other EVP tasks (*n* = 5 studies). Additionally, exploratory mixed-effect model meta-regression analyses were performed to investigate potential moderating effects of demographic and clinical variables on the associations between EVP, SC, and FOs. Due to incomplete information on moderator values in some studies, each moderator was assessed separately.

A meta-mediation analysis was conducted to examine whether SC mediates the relationship between EVP and FOs, using the ‘metafor’ (version 4.6–0) and ‘lavaan’ (version 0.6–17) packages in R (version 4.3.2). The analysis incorporated a two-stage meta-analytic structural equation modelling (MASEM) approach. First, correlations were pooled across studies using random effect models to account for between-study heterogeneity. The pooled correlation matrix was then used to fit the proposed mediation model. Maximum likelihood (ML) estimation was employed to evaluate the mediation model, which included estimating the direct effects of EVP on FOs, EVP on SC, and SC on FOs. The indirect effect of EVP on FOs through SC was calculated by multiplying the path coefficients from EVP to SC and from SC to FOs. Additionally, the proportion of the total effect mediated by SC was computed.

## 3 Results

### 3.1 Study selection and characteristics

**Fig. 1:**
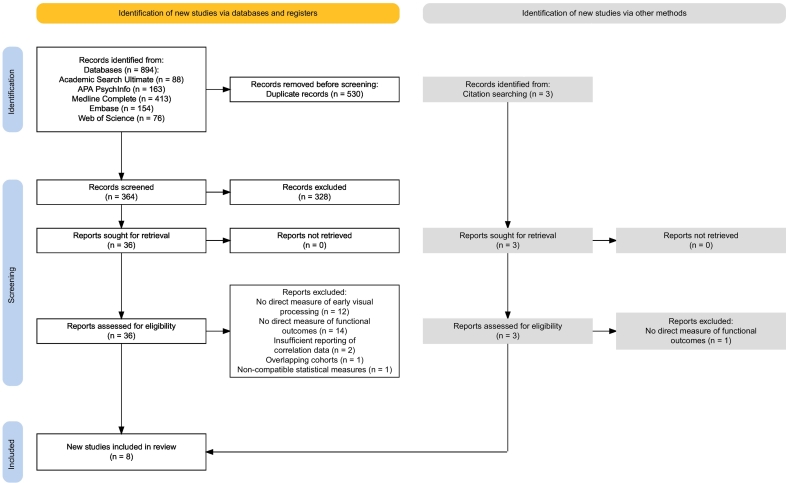
PRISMA flow diagram illustrating the selection process of studies for inclusion in the systematic review and meta-analysis, including reasons for exclusion at each stage.

Following a systematic literature search and screening process, twelve independent studies were identified for inclusion (see Fig. 1). Three subsequent exclusions were made due to the use of Spearman's rho instead of Pearson's *r*, making it incompatible with the other studies (Keane et al., 2014), and lack of reporting on non-significant associations (Kim et al., 2005; Yang et al., 2013). Two studies with identical overlapping samples presenting the same cognition-outcome pairs were identified and averaged (Horton and Silverstein, 2008; Horton and Silverstein, 2007). Ultimately, eight studies met all criteria for inclusion. These studies collectively encompassed 764 participants, with an average age of 42.69 years (SD = 3.78) and a proportion of males of 72.24 % (SD = 13.24). Ethnicity information was available in only two studies, with an average representation of 53.6 % (SD = 10.46) white participants. Information on education was reported in five studies, averaging 13.16 years (SD = 0.59). The average duration of illness among participants was 20.24 years (SD = 4.13), as reported in seven studies. The majority of participants were on antipsychotic medications (95.03 %, SD = 4.55), though specific dosage information was available for only three studies, averaging 844.18 mg (SD = 367.1) of chlorpromazine equivalent per day. Most participants were diagnosed with schizophrenia (91.73 %), with the remainder having a diagnosis of schizoaffective disorder. The characteristics of the included studies and the outcome measures are detailed in Table 2.

**Table 2:** Characteristics and outcomes of included studies evaluating the relationships between early visual processing, social cognition, and functional outcomes in schizophrenia spectrum disorders.

Authors	Participants N	Age (yrs) Mean (SD)	Male N, (%)	White N, (%)	Education (yrs) Mean (SD)	Diagnosis	Illness Duration (yrs) Mean (SD)	Antipsychotic Use (%)	Chlorpromazine Daily Equivalent (mg) Mean (SD)	Study Setting	Country	Quality Assessment Score	Main Outcomes	Effect Size (r)
Brittain et al., 2010	64	41.9 (11.1)	34 (53.1)	–	14.2 (3.2)	100 % SZ	18.41	92.2	461.95 (381.61)	outpatient	UK	8	EVP-FO EVP-SC SC-FO	0.14 0.23 0.30
Butler et al., 2005	33	37.1 (1.7)	26 (78.8)	–	–	81.8 % SZ, 18.2 % SZA	14.5 (1.7)	100	1194.0 (91.7)	in- and outpatient	USA	7	EVP-FO	0.44
Green et al., 2012a	191	46.6 (9.8)	129 (67.5)	–	12.7 (1.8)	90.6 % SZ, 9.4 % SZA	24.2 (11.3)	93.7	–	outpatient	USA	8	EVP-FO EVP-SC SC-FO	0.12 0.18 0.31
Herrera et al., 2021	58	38.9 (10.0)	46 (79.3)	–	–	84.5 % SZ, 15.5 % SZA	16.3 (9.1)	98.3	876.6 (689.7)	–	USA	6	EVP-FO	0.08
Horton & Silverstein, 2008	65	46 (9.1)	43 (66.2)	30 (46.2)	–	76.9 % SZ, 23.1 % SZA	26 -	–	–	–	USA	8	EVP-FO EVP-SC	0.36 0.33
Rassovsky et al., 2011	174	44.5 (9.9)	144 (83)	–	12.9 (1.7)	100 % SZ	21.1 (11.2)	98	–	out-patient	USA	7	EVP-FO EVP-SC SC-FO	0.16 0.25 0.23
Sergi et al., 2006	75	46.7 (9.5)	69 (92)	–	13.0 (1.8)	100 % SZ	21.2 (11.0)	88	–	–	USA	5	EVP-FO EVP-SC SC-FO	0.12 0.34 0.27
Sheffield et al., 2014	104	39.8 (11.9)	60 (58)	63 (61)	13.0 (3.9)	–	–	–	–	–	USA	7	EVP-FO	0.08

*Note*. SZ = Schizophrenia. SZA = Schizoaffective Disorder. EVP = Early Visual Processing. SC = Social Cognition. FO = Functional Outcomes.

### 3.2 Early visual processing and functional outcomes

**Fig. 2:**
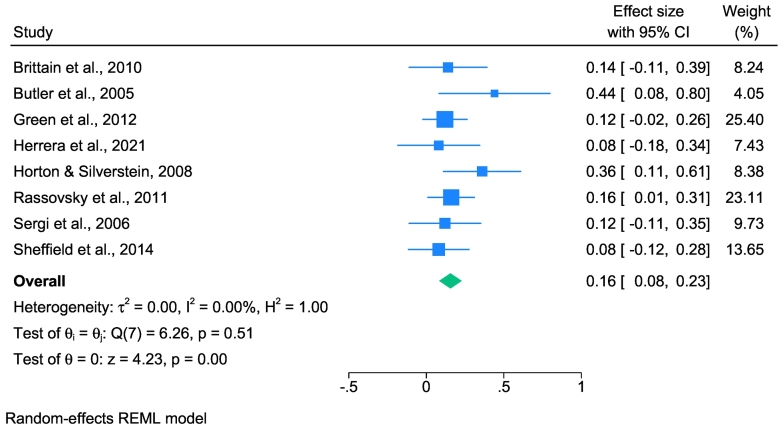
Forest plot displaying estimated effect sizes and 95 % confidence intervals obtained from the meta-analysis of associations between early visual processing and functional outcomes in schizophrenia spectrum disorders. *Note.* REML, Restricted Maximum Likelihood. Effect sizes are displayed as the correlation coefficient *r* to facilitate interpretation.

Eight studies investigated the relationship between EVP and FOs (see Fig. 2). The analysis revealed a sample-weighted average effect size of *û*_p_ = 0.16 (95 % CI: 0.087 to 0.231), indicating a small but statistically significant association (*z* = 4.33, *p* < .001). There was no heterogeneity across studies (*I*^*2*^ = 0.00), suggesting consistency in the observed effects.

Subgroup analyses were conducted to examine the impact of different EVP assessment methods: visual masking tasks (Group 1) and other EVP tasks (Group 0). Group 0, which included five studies, had a sample-weighted average effect size of *û*_p_ = 0.20 (95 % CI: 0.059 to 0.347), indicating a significant effect (*p* = .01) and low to moderate heterogeneity (*I*^*2*^ = 37.16). Similarly, Group 1, comprising three studies, showed a significant effect with a sample-weighted average effect size of *û*_p_ = 0.14 (95 % CI: 0.042 to 0.231; *p* < .001) and no observed heterogeneity (*I*^*2*^ = 0.00). However, there were no significant differences between the two groups (*p* = .45), indicating that the type of EVP assessment did not significantly affect the overall effect size (see Supplementary Fig. S2).

### 3.3 Early visual processing and social cognition

**Fig. 3:**
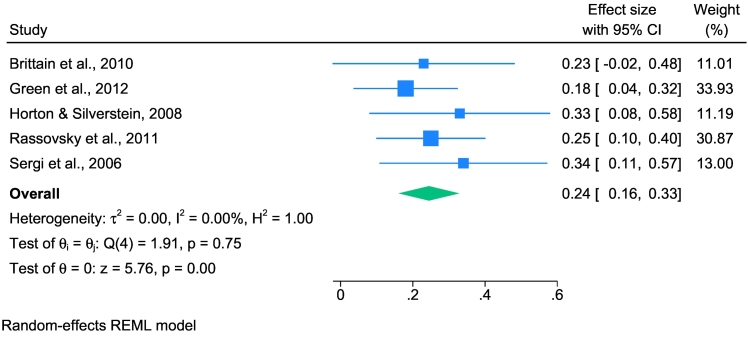
Forest plot displaying estimated effect sizes and 95 % confidence intervals obtained from the meta-analysis of associations between early visual processing and social cognition in schizophrenia spectrum disorders. *Note.* REML, Restricted Maximum Likelihood. Effect sizes are displayed as the correlation coefficient *r* to facilitate interpretation.

The analysis of the relationship between EVP and SC included five studies (see Fig. 3). The sample-weighted average effect size was *û*_p_ = 0.25 (95 % CI: 0.167 to 0.334), reflecting a moderate significant effect with no heterogeneity (*I*^*2*^ = 0.00).

Subgroup analysis revealed no significant variance in effect sizes between the groups using different EVP tasks (*Q*_*b*_ = 0.23, *p* = .631), suggesting consistent effects across different EVP assessment methods (see Supplementary Fig. S3).

### 3.4 Social cognition and functional outcomes

**Fig. 4:**
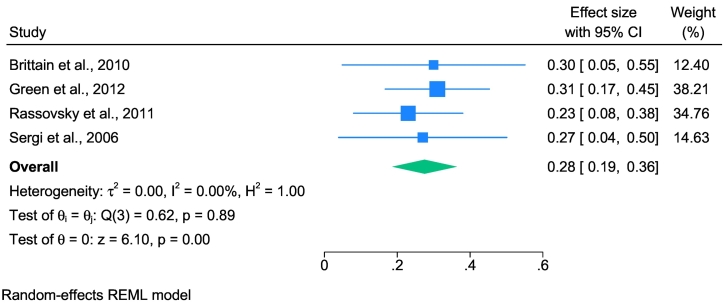
Forest plot displaying estimated effect sizes and 95 % confidence intervals obtained from the meta-analysis of associations between social cognition and functional outcomes in schizophrenia spectrum disorders. *Note.* REML, Restricted Maximum Likelihood. Effect sizes are displayed as the correlation coefficient *r* to facilitate interpretation.

The relationship between SC and FOs was analysed across four studies. The sample-weighted average effect size was moderate (*û*_p_ = 0.28; 95 % CI: 0.194 to 0.371) and there was no heterogeneity (*I*^*2*^ = 0.00; see Fig. 4).

### 3.5 Moderator analyses

Moderator analyses were conducted to determine the influence of demographic and clinical characteristics on the relationships between EVP, SC, and FOs. Age, gender, education, illness duration, antipsychotic treatment, daily chlorpromazine equivalents, and methodological quality of the study were included in the meta-regression models. No significant moderating effects were found for the associations between EVP, SC, and FOs, indicating that the observed effect sizes were largely stable across different demographic and clinical characteristics. Full results of these analyses are presented in Supplementary Tables S6-S8.

### 3.6 Mediation analysis

**Fig. 5:**
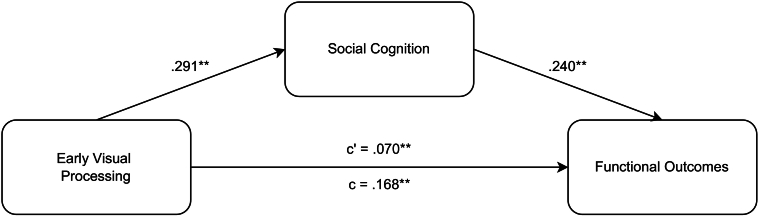
Meta-mediation analysis examining the relationships between early visual processing, social cognition, and functional outcomes in schizophrenia spectrum disorders. *Note.* c = direct effect, c' = mediated (i.e., partial) effect. **p < .001.

A mediation analysis was conducted to investigate whether SC mediates the relationship between EVP and FOs (see Fig. 5). The analysis revealed a significant direct effect of EVP on FOs (*β* = 0.168, SE = 0.036, *z* = 4.718, *p* < .001), and a significant effect of EVP on SC (*β* = 0.291, SE = 0.035, *z* = 8.403, *p* < .001). Additionally, SC was significantly associated with FOs (*β* = 0.240, SE = 0.036, *z* = 6.738, *p* < .001). The analysis also confirmed the mediating role of SC, with a significant indirect effect of EVP on FOs through SC (*β* = 0.070, SE = 0.013, *z* = 5.257, *p* < .001). Moreover, the total effect of EVP on FOs, incorporating both direct and mediated effects, was significant (*β* = 0.238, SE = 0.035, *z* = 6.781, *p* < .001). However, due to the limited number of studies (*n* = 4), caution is advised in interpreting the strength and significance of these findings.

### 3.7 Publication bias

Publication bias was evaluated using Egger's regression test along with funnel plot analyses for each meta-analysis (see Supplementary Figs. S4-S6). The results indicated no presence of publication bias. However, it is important to note that Egger's test might have limited ability to identify bias in analyses with a small number of studies (Harrer et al., 2021). To support the findings from Egger's regression tests, funnel plots were reviewed, showing no significant asymmetry. Overall, the analyses did not reveal any evidence that the estimated effects were influenced by publication bias.

## 4 Discussion

This systematic review and meta-analysis synthesised evidence on the relationships between EVP, SC, and FOs in individuals with SSDs. By integrating data from eight studies and conducting a meta-mediation analysis, we explored how EVP and SC deficits may contribute to functional impairments, offering insights for both theoretical frameworks and clinical interventions.

### 4.1 The relationship between early visual processing and functional outcomes

Our meta-analysis revealed a small but statistically significant association between EVP and FOs (*r* = 0.16), indicating that early perceptual impairments are linked to functional limitations in individuals with SSDs (Kim et al., 2005; Li et al., 2020; Green et al., 2012a). Deficits in EVP can make it more difficult to process and interpret visual information efficiently, which may contribute to challenges in daily tasks such as reading instructions (Revheim et al., 2006) or safely navigating while driving (Keay et al., 2009). These functional limitations could extend to difficulties in recognising and responding to visual social cues, which play a crucial role in effective social interactions (Sergi and Green, 2003).

However, it is important to note that our conceptualisation of EVP included multiple distinct processes, including contrast sensitivity, visual masking, span of apprehension, and visual integration. These functions operate at different hierarchical levels of visual processing and engage distinct neural mechanisms. For instance, contrast sensitivity primarily involves early-stage visual processing at the retinal level (Silverstein and Rosen, 2015). In contrast, visual masking, especially backward masking, requires more complex visual processing and feedback mechanisms at the cortical level, including involvement of subcortical structures like the thalamus and cortical regions such as the lateral occipital area (Green et al., 2005b). Span of apprehension, which relies on short-term memory and attentional control, along with visual integration reflect more advanced stages of processing. Hence, grouping these diverse processes under the broad term “EVP” may obscure the specific contributions of each function to FOs. This limits our ability to draw precise conclusions about how different aspects of EVP relate to SC and FOs in non-affective psychotic disorders.

Due to limited data, we were also unable to individually examine the impact of EVP on specific FO domains, such as social functioning, work productivity, independent living, and self-care. Treating these domains as a single entity oversimplifies how EVP deficits might differentially affect each area of functioning. Prior research indicates that while these domains are linked, they might follow distinct biosocial pathways (Brekke et al., 1997), with some being more reliant on visuospatial skills and others on visuomotor or verbal processing (Brekke and Long, 2000). To fully understand the associations between EVP deficits and FOs, further research is needed to gather sufficient data to analyse EVP tasks and FO domains individually.

Furthermore, the FO measures included in this meta-analysis varied in their methodological approaches. Performance-based FO measures provide more objective indicators of real-world functional ability, as they require individuals to complete structured tasks that reflect functional demands and are closely tied to cognitive and sensory processing (Elliott and Fiszdon, 2014; Harvey et al., 2007). In contrast, self-report measures reflect individuals' subjective perceptions of their functioning, which may be influenced by factors such as insight, mood, or cognitive biases (Elliott and Fiszdon, 2014; Harvey et al., 2007; Harvey, 2011). In line with this, research has shown that self-reported everyday functioning in SSDs is weakly related to performance-based measures (e.g., *r* = 0.06), whereas clinician-rated FOs exhibit stronger correlations with performance-based assessments (e.g., *r* = 0.42) (Bowie et al., 2007; Keefe et al., 2006; Sabbag et al., 2011). Similarly, high-contact clinicians have been found to generate FO ratings that significantly correlate with cognitive ability measures, such as the MATRICS Consensus Cognitive Battery (MCCB; *r* = 0.36–0.46), while self-reports and informant reports from non-clinicians often show weak or nonsignificant relationships with cognitive and functional capacity (e.g., *r* = 0.06–0.15) (Bowie et al., 2007; Keefe et al., 2006; Sabbag et al., 2011).

Among the studies included in our meta-analysis, two studies used a performance-based FO measure (Green et al., 2012a; Sheffield et al., 2014), whereas the majority relied on clinician-administered semi-structured interviews (Butler et al., 2005; Herrera et al., 2021; Brittain et al., 2010; Rassovsky et al., 2011; Sergi et al., 2006) or informant reports from case managers (Horton and Silverstein, 2008). Sheffield et al. (2014) was the only study that combined multiple FO assessment methods, including performance-based, self-report, and informant ratings.

Given that most studies in our meta-analysis relied on clinician-administered measures rather than self-reports or non-clinician informants, it is likely that the FO assessments captured functional ability in a more objective manner that aligns closely with performance-based evaluations. However, future research should further explore how different FO assessment types relate to EVP deficits and their broader impact on FOs.

### 4.2 The relationship between early visual processing and social cognition

We found a significant, moderate relationship between EVP and SC (*r* = 0.25). Prior research suggests that impairments in EVP may impede the accurate perception and processing of visual information crucial for SC. This disruption could contribute to difficulties in interpreting social cues, inferring mental states, and understanding emotions, leading to broader social dysfunction and difficulties in social interactions (Fett et al., 2011; Halverson et al., 2019). For instance, individuals with EVP deficits may struggle to process subtle changes in facial expressions or body movements, leading to misunderstandings in social interactions. This difficulty can hinder the recognition of social hierarchies, group dynamics, or emotional states, resulting in inappropriate social responses and inaccurate social judgments. Moreover, EVP impairments might cause misinterpretations of gaze or facial expressions, leading to incorrect assumptions about others' intentions or feelings. These challenges can complicate the prediction of behaviour and understanding of social interactions, which are crucial for effective communication and relationship-building.

An alternative explanation, however, is that EVP and SC deficits are not entirely hierarchical but may arise from a shared underlying neural dysfunction, such as an excitation/inhibition imbalance (Anticevic and Lisman, 2017). Research shows that disruptions in neurotransmitter balance, particularly involving GABAergic and glutamatergic systems, affect both sensory and higher-order cognitive functions (Foss-Feig et al., 2017; Javitt, 2023; Mehta et al., 2014). This suggests that EVP and SC impairments might not follow a sequential pathway but may instead reflect common neural deficits.

Again, our analysis was limited by insufficient data to perform subgroup analyses on individual SC domains, limiting our understanding of domain-specific effects of EVP deficits on SC. For instance, while EVP deficits may impair emotion perception by making it difficult to accurately identify facial expressions, they might affect ToM differently by hindering the interpretation of subtle visual cues, such as gaze direction or gestures, which could help infer others' intentions. Further research is needed to understand these potentially domain-specific associations in a more nuanced way.

### 4.3 The relationship between social cognition and functional outcomes

In line with previous research, we found a moderate, significant association between SC deficits and poorer FOs (*r* = 0.28). This aligns with the broader literature suggesting that SC impairments are linked to social and occupational difficulties in individuals with SSDs (Green et al., 2015; Sergi et al., 2006). For instance, difficulties in understanding social cues, interpreting emotions, and engaging in effective communication can hinder an individual's ability to function in social and occupational settings, potentially limiting job opportunities and reducing social integration. Moreover, SC deficits may impair independent living by reducing the ability to interpret and respond to social signals necessary for daily interactions, potentially leading to social withdrawal, isolation, and further functional decline.

While our study could not differentiate between specific FO domains due to limited data, prior research suggests that these relationships may vary across different functional areas (Halverson et al., 2019; Lemmers-Jansen et al., 2023). Notably, the strength of the observed SC–FO relationship may also be influenced by how FOs are assessed. For instance, Sheffield et al. (2014) found that performance-based FO measures were more strongly associated with cognitive abilities, whereas self-report and informant-based assessments demonstrated weaker or inconsistent relationships. Given that self-reported and informant-rated FO measures may be affected by subjective biases such as mood, insight, and cognitive distortions (Harvey, 2011; Bowie et al., 2007; Keefe et al., 2006), the extent to which SC predicts real-world functioning could vary across studies depending on the assessment method used. Future research should consider the impact of FO measurement type when evaluating the role of SC in functional impairments, as performance-based assessments may provide a more accurate reflection of the real-world consequences of SC deficits in SSDs.

### 4.4 Mediation analysis

Our meta-mediation analysis revealed a small but significant indirect effect of SC (*c’* = 0.07), indicating that SC partially mediates the relationship between EVP and FOs. However, the direct effect of EVP on FOs (*c* = 0.17) remained significant, suggesting that additional factors contribute to functional impairments. Previous studies have identified higher-order non-social cognitive deficits, such as working memory, as relevant contributors (Haenschel et al., 2007; González-Ortega et al., 2013; Zaragoza Domingo et al., 2015). Moreover, negative symptoms, particularly motivational deficits like avolition and anhedonia, are well-established predictors of functional impairments (Breier et al., 1991; Fenton and McGlashan, 1994). Defeatist performance beliefs, which are overly generalised negative beliefs about one's ability to succeed in tasks, have also been shown to mediate the relationship between EVP deficits and poorer FOs (Green et al., 2012a; Rassovsky et al., 2011). According to Beck's model of negative symptoms, reduced abilities can lead to discouraging experiences, which in turn foster defeatist beliefs that diminish motivation, perpetuating a cycle of negative symptoms and functional decline (Beck et al., 2009; Grant and Beck, 2009; Horan et al., 2010). These factors, while influential, do not fully account for the variance in FOs, underscoring the need for further research to identify additional mechanisms that mediate the relationship between EVP deficits and functional impairments.

The neural mechanisms linking EVP, SC, and FOs likely involve disruptions in local and distributed brain networks, with EVP deficits often associated with abnormalities in EVP regions, such as the primary visual cortex and associated extrastriate areas (Silverstein and Keane, 2011; Butler et al., 2008). These disruptions may impair higher-order visual integration and perceptual organisation, which are critical for accurately interpreting social cues and complex visual scenes (Green et al., 2012a). The dysfunctions in these early visual areas may impair neural circuits involved in SC, particularly those associated with the superior temporal sulcus, fusiform gyrus, and medial prefrontal cortex, which are crucial for interpreting facial expressions, eye gaze, and social intentions (Phillips et al., 2015; Pinkham et al., 2007). Moreover, top-down modulation from higher-order brain areas, such as the prefrontal cortex, plays a significant role in shaping how early visual information is processed (Rauss et al., 2011). For example, focused attention can enhance the processing of specific visual stimuli, making them more salient and easier to detect (Desimone and Duncan, 1995). Predictions about incoming sensory information, based on past experiences, can also shape how early visual information is processed, leading to faster and more efficient recognition of expected stimuli (Summerfield and Egner, 2009). Disruptions in these top-down processes in SSDs could contribute to the observed deficits in EVP, compounding the sensory deficits characteristic of the disorder (Javitt, 2009b).

### 4.5 Implications

Clinically, our findings suggest that interventions targeting both EVP and SC deficits may offer a more effective strategy for improving FOs in individuals with SSDs. Research indicates that remediating visual processing deficits through targeted visual training can lead to improvements in basic perceptual skills, such as visual backward masking, motion perception, contrast sensitivity, visual search efficiency, visual acuity, and perceptual organisation (Contreras et al., 2018; Demmin et al., 2019). These improvements in EVP have been linked to improved SC abilities, which in turn have shown promise in leading to better FOs (Scoriels et al., 2022). Similarly, SC training programmes have demonstrated efficacy in improving facial affect recognition, mentalising, and social perception (Horan et al., 2009; Kurtz et al., 2016; Kurtz and Richardson, 2012), which have been associated with better FOs (Kurtz and Richardson, 2012).

While EVP and SC interventions have typically been studied in isolation, research suggests that combining cognitive and social-cognitive training may yield greater benefits. For instance, auditory-based cognitive training has been shown to improve verbal memory and global cognition by strengthening both bottom-up sensory processing and top-down cognitive functions (Adcock et al., 2009). Building on this, Fisher et al. (2017) found that supplementing neurocognitive training with SC exercises resulted in greater gains in prosody identification and reward processing, key factors influencing social motivation and FOs. These findings suggest that a multi-domain training approach that integrates EVP and SC interventions could provide a more comprehensive strategy for enhancing real-world functioning in SSDs.

### 4.6 Limitations and future directions

While our analyses provide valuable insights, several limitations in the existing research should be acknowledged. The small number of included studies limited our ability to conduct subgroup analyses, preventing us from exploring how specific SC and FO domains may be differentially affected by EVP deficits. Additionally, variability in FO assessment methods (e.g., performance-based measures vs. self-reports) may capture distinct aspects of functioning, complicating direct comparisons. Future studies should aim to disentangle these nuances to better understand the mechanisms linking EVP, SC, and FOs.

The limited demographic data in the included studies also restricted our ability to perform moderator analyses on ethnicity, and subgroup analyses on education and medication use, which limits the insights gained from our meta-regression. Medication use, in particular, has been shown in previous research to significantly impact cognitive functioning, yet it was not consistently controlled for across studies. Future studies should include larger, more ethnically and culturally diverse samples and consider the role of antipsychotic medication dosages and treatment adherence in influencing cognition and FOs.

Another important consideration is the chronicity of the included samples, as most participants had a long illness duration, averaging 20 years. It is possible that the associations under investigation vary across illness stages. However, research indicates that EVP deficits, such as impairments in visual masking and contrast sensitivity, emerge early in the course of SSDs and may even predate illness onset, serving as potential vulnerability markers (Perez et al., 2012; Schwarzer et al., 2022; Yeap et al., 2008). These deficits appear relatively stable over time, with similar impairments observed in both first-episode psychosis (FEP) and chronic schizophrenia (Yeap et al., 2008).

Similarly, SC deficits seem to persist across illness stages, though longitudinal research remains limited. A cross-sectional study by Green et al. (2012b) found no significant differences in SC performance between early and chronic schizophrenia, suggesting that these impairments do not significantly worsen over time. Longitudinal studies have also shown relative stability in SC, with Horan et al. (2012) reporting no significant decline over one year in early schizophrenia, and McCleery et al. (2016) observing stable SC performance over a five-year follow-up in both recent-onset and chronic patients.

FOs also appear to remain relatively stable throughout the illness rather than showing progressive decline or notable improvement. A cross-sectional comparison of early-stage and chronic schizophrenia patients found no significant differences in functional impairments (Costa et al., 2014). Longitudinal studies similarly indicate that once functional impairments are present, they tend to persist over decades without substantial changes (Harrow et al., 2017; Harrow and Jobe, 2007; Velthorst et al., 2017; Wiersma et al., 2000).

Taken together, these findings suggest that impairments in EVP, SC, and FOs are relatively stable over the course of SSDs, reinforcing the importance of early interventions to mitigate long-term functional impairment. Future research should further explore whether specific subgroups may follow different trajectories, as well as potential mechanisms that could influence variability in these domains over time.

Although our findings support the plausibility of an EVP → SC → FO pathway, the cross-sectional nature of the included studies precludes causal conclusions. Experiments using temporally sensitive neuroimaging techniques, such as EEG, could help clarify the temporal sequence of these processes. For instance, early visual components like P1 and N170 reflect basic visual processing and could provide insights into how disruptions in EVP temporally relate to SC impairments.

However, it is also possible that these relationships are bidirectional rather than strictly hierarchical. For example, SC impairments may influence how individuals allocate attention to visual information, thereby potentially exacerbating EVP deficits over time (Rauss et al., 2011; Zani and Proverbio, 2012). Additionally, shared underlying mechanisms, such as disruptions in excitatory/inhibitory balance or global neurodevelopmental abnormalities, may contribute to both EVP and SC impairments, leading to their observed associations with FOs (Foss-Feig et al., 2017; Javitt, 2023). Future research should explore these alternative explanations to provide a more comprehensive understanding of the interconnections between these cognitive domains.

## 5 Conclusion

This systematic review and meta-analysis synthesises existing evidence linking EVP deficits to functional impairments in individuals with SSDs, potentially through their impact on SC. However, these findings should be interpreted with caution given the small number of available studies and methodological variability. Future research should aim to refine these models by considering how the nature of the stimuli and the presence of top-down influences might affect the relationships between EVP, SC, and FOs. Addressing these unanswered questions will improve our understanding of the cognitive pathways in SSDs and contribute to the development of more effective therapeutic strategies.

## CRediT authorship contribution statement

**Akke Ganse-Dumrath:** Writing – review & editing, Writing – original draft, Visualization, Software, Formal analysis, Data curation, Conceptualization. **Anya Chohan:** Validation, Data curation. **Steven Samuel:** Writing – review & editing. **Paul Bretherton:** Writing – review & editing. **Corinna Haenschel:** Writing – review & editing, Supervision, Conceptualization. **Anne-Kathrin Fett:** Writing – review & editing, Supervision, Formal analysis, Conceptualization.

## Funding sources

AGD and AC are supported by a 10.13039/100007566City, University of London PhD studentship.

## Declaration of competing interest

The authors declare the following financial interests/personal relationships which may be considered as potential competing interests: Co-author serves as a member of the editorial board for Schizophrenia Research: Cognition - A.-K.F. If there are other authors, they declare that they have no known competing financial interests or personal relationships that could have appeared to influence the work reported in this paper.

## Appendix A Supplementary data

Supplementary material Image 1

## Footnotes

2 **Abbreviations: SSDs:** Schizophrenia Spectrum Disorders; **EVP**: Early Visual Processing; **SC**: Social Cognition; **FOs**: Functional Outcomes.
