# Supplementary material for: Systematic review and meta-analysis of early visual processing, social cognition, and functional outcomes in schizophrenia spectrum disorders
Source: Schizophr Res Cogn. 2025 Feb 15;40:100351. doi: 10.1016/j.scog.2025.100351 (PMC11872129; doi:10.1016/j.scog.2025.100351)
Supplement: Supplementary file 1 — Supplementary material [file mmc1.docx]

**Supplementary Material**

**Figures**

**Supplementary Figure S1.** Decision flowchart for the article screening process.

**Supplementary Figure S2**. Forest plot displaying estimated effect sizes and 95% confidence intervals obtained from the meta-analysis of associations between early visual processing (EVP) and functional outcomes in schizophrenia spectrum disorders divided into a subgroup of studies using visual masking tasks (Group 1) and other EVP tasks (Group 0).


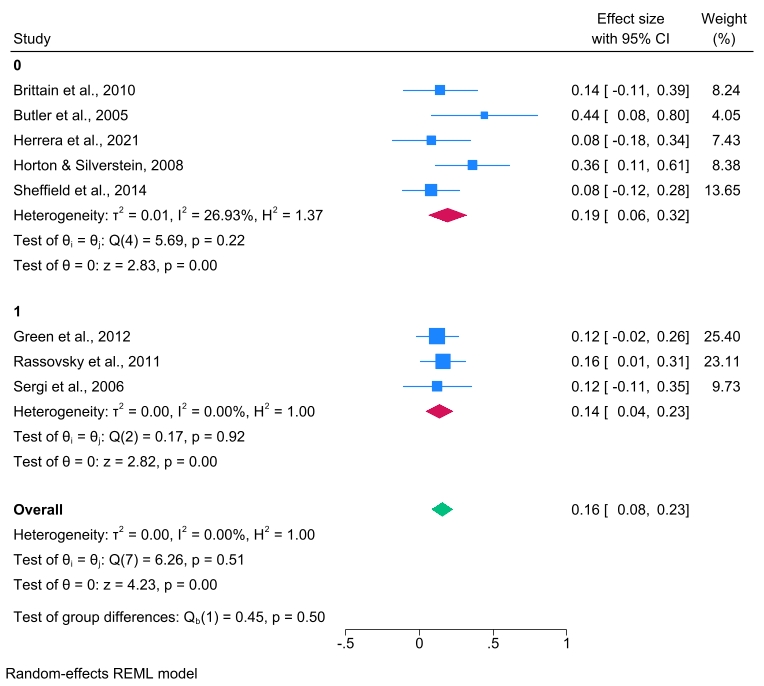


*Note.* REML, Restricted Maximum Likelihood. Effect sizes are displayed as the correlation coefficient *r* to facilitate interpretation.

**Supplementary Figure S3.** Forest plot displaying estimated effect sizes and 95% confidence intervals obtained from the meta-analysis of associations between early visual processing (EVP) and social cognition in schizophrenia spectrum disorders divided into a subgroup of studies using visual masking tasks (Group 1) and other EVP tasks (Group 0).


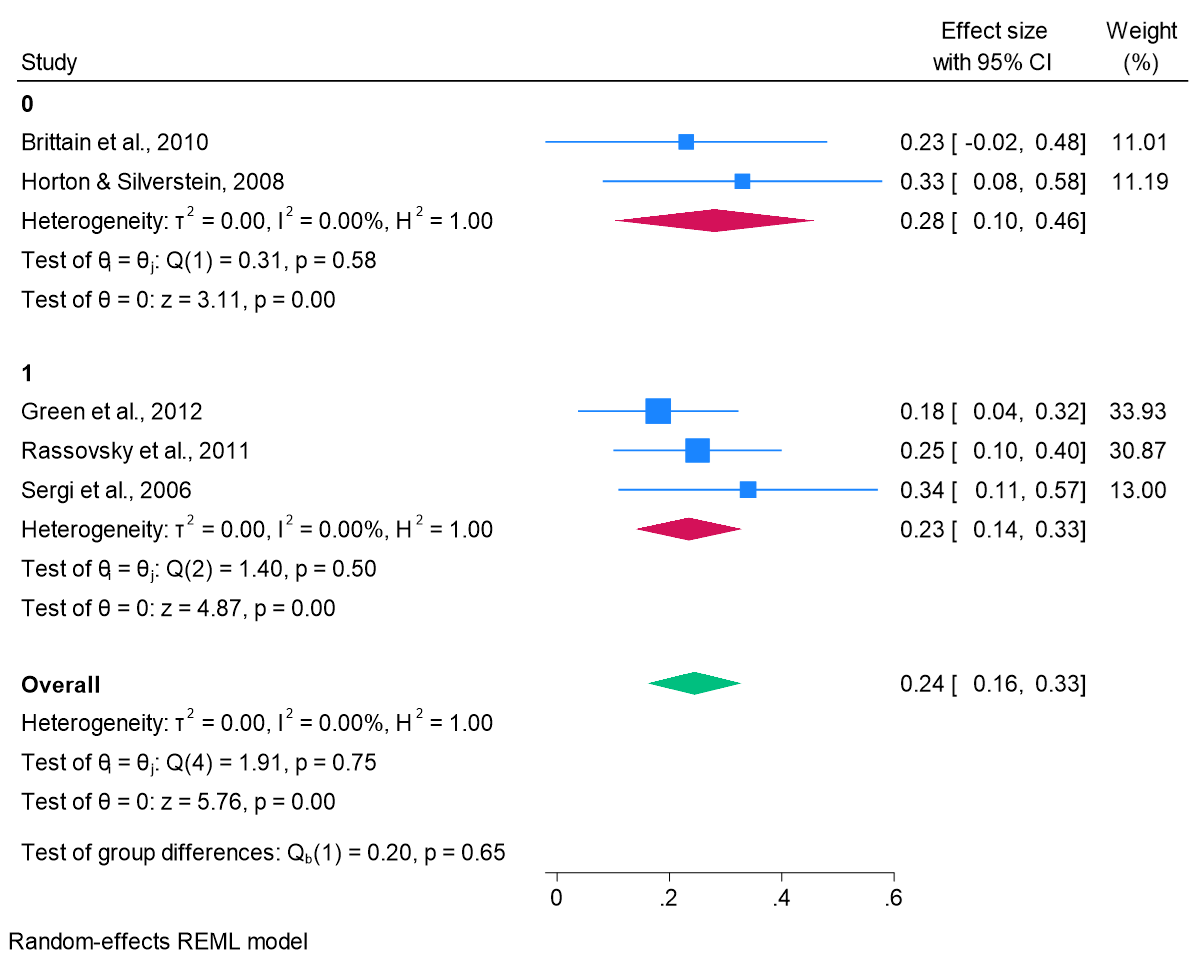


*Note.* REML, Restricted Maximum Likelihood. Effect sizes are displayed as the correlation coefficient *r* to facilitate interpretation.

**Supplementary Figure S4.** Funnel plot for the relationship between early visual processing and functional outcomes in schizophrenia spectrum disorders.


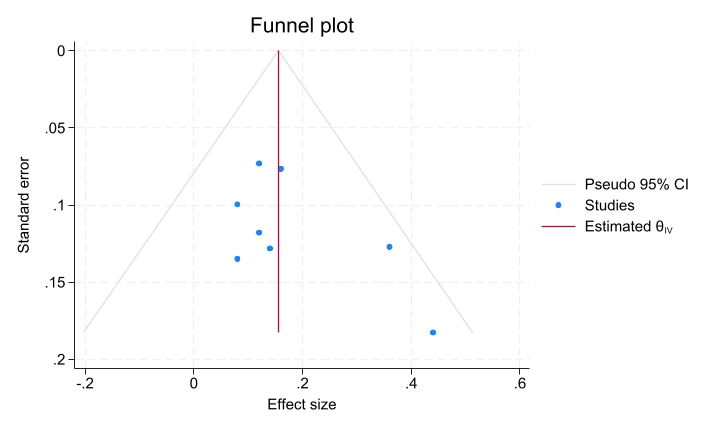


**Supplementary Figure S5.** Funnel plot for the relationship between early visual processing and social cognition in schizophrenia spectrum disorders.


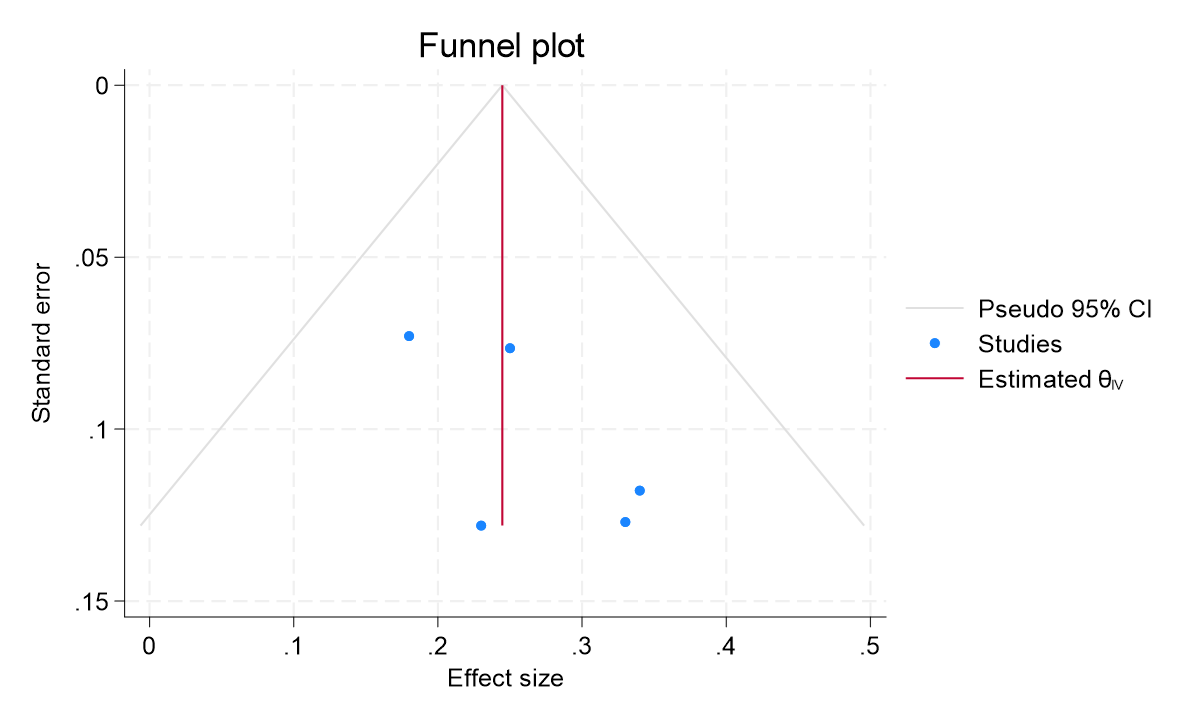


**Supplementary Figure S6.** Funnel plot for the relationship between social cognition and functional outcomes in schizophrenia spectrum disorders.


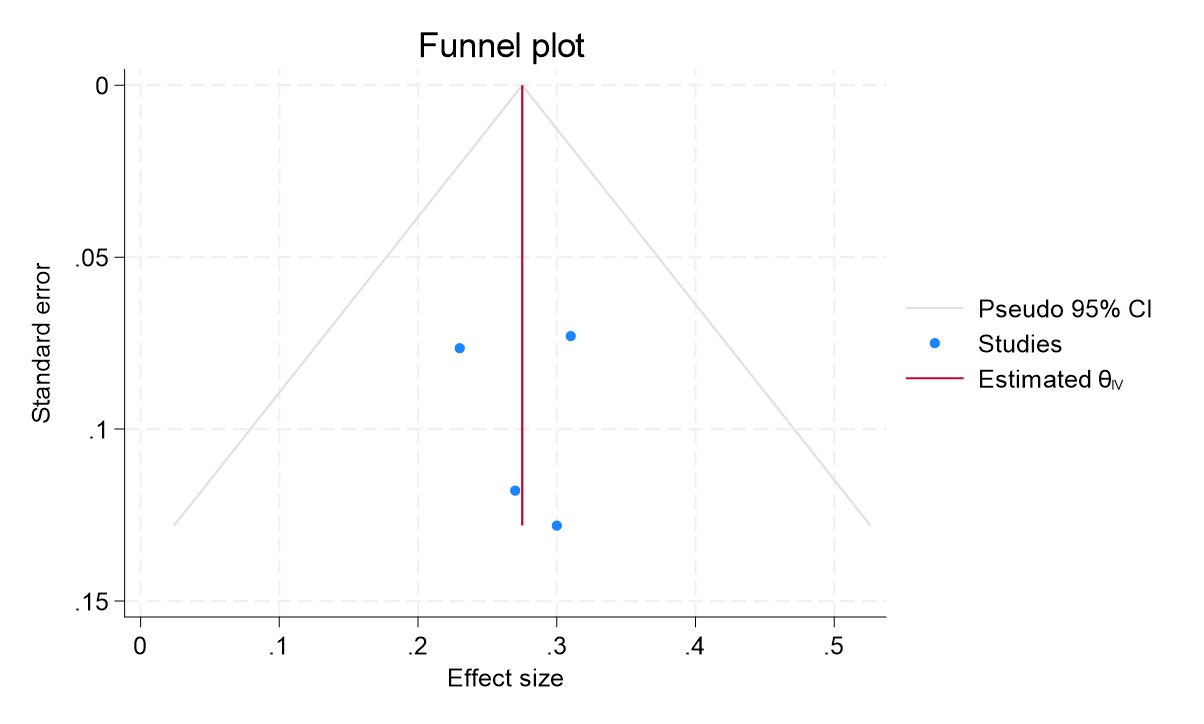


**Tables**

**Supplementary Table S1.** Search strategy employed in the *Academic Search Ultimate* database using the *EBSCOhost* platform.

| **Search ID** | **Search Terms** | **Search Options** |
| --- | --- | --- |
| S1 | (DE "SCHIZOPHRENIA") OR (DE "SCHIZOTYPAL personality disorder") OR (DE "PSYCHOSES") OR (DE "SCHIZOPHRENIFORM disorder") | Expanders - Apply equivalent subjects  Search modes - Boolean/Phrase |
| S2 | AB schizo* or "schizophrenia spectrum disorder*" or "non-affective psychos*" or "schizotypal personality disorder" or "psychotic disorder*" or "schizophreniform disorder" or "schizoaffective disorder" or "brief psychotic disorder" or psychos?s or "delusional disorder" | Expanders - Apply equivalent subjects  Search modes - Boolean/Phrase |
| S3 | S1 OR S2 | Expanders - Apply equivalent subjects  Search modes - Boolean/Phrase |
| S4 | DE "VISUAL perception" | Expanders - Apply equivalent subjects  Search modes - Boolean/Phrase |
| S5 | AB "early visual processing" or "basic visual processing" or "visual process*" or "visual percept*" or "visual function*" or "visual abnormalit*" or "visuo-perceptual deficit*" or "visual information" | Expanders - Apply equivalent subjects  Search modes - Boolean/Phrase |
| S6 | S4 OR S5 | Expanders - Apply equivalent subjects  Search modes - Boolean/Phrase |
| S7 | (DE "THEORY of mind") OR (DE "SOCIAL cues") OR (DE "EMPATHY") OR (DE "EMOTION recognition") OR (DE "ATTRIBUTION (Social psychology)") | Expanders - Apply equivalent subjects  Search modes - Boolean/Phrase |
| S8 | AB "social cogniti*" or "social percept*" or "social knowledge" or "social judg#ment" or "social cue*" or "theory of mind" or "ToM" or mentali* or "mind reading" or empathy or "emotion recognition" or "affect recognition" or "emotion perception*" or "affect perception" or attribution or "attributional bias" | Expanders - Apply equivalent subjects  Search modes - Boolean/Phrase |
| S9 | S7 OR S8 | Expanders - Apply equivalent subjects  Search modes - Boolean/Phrase |
| S10 | (DE "QUALITY of life") OR (DE "LIFE satisfaction") OR (DE "SOCIAL interaction") OR (DE "SOCIAL adjustment") OR (DE "SOCIAL participation") OR (DE "LIFE skills") | Expanders - Apply equivalent subjects  Search modes - Boolean/Phrase |
| S11 | AB "functional outcome" or "social outcome" or "social functioning" or "work functioning" or "occupational functioning" or "daily functioning" or "vocational functioning" or "quality of life" or "life satisfaction" or "social relationships" or "social integration" or "social behavio#r" or "social adjustment" or "social dysfunction" or "independent living skills" or "skills of daily living" or "life skills" or "social skill*" or "social interaction" or "social participation" or "social-cognitive performance" or "psychosocial outcome*" or "social adapt*" or "social disabilit*" or "social competenc*" | Expanders - Apply equivalent subjects  Search modes - Boolean/Phrase |
| S12 | S10 OR S11 | Expanders - Apply equivalent subjects  Search modes - Boolean/Phrase |
| S13 | S3 AND S6 AND S9 AND S12 | Limiters - Peer Reviewed; Publication Date: 19770101-20231131; Publication Type: Academic Journal; Language: English  Expanders - Apply equivalent subjects  Search modes - Boolean/Phrase |
| S14 | S3 AND S6 AND S12 | Limiters - Peer Reviewed; Publication Date: 19770101-20231131; Publication Type: Academic Journal; Language: English  Expanders - Apply equivalent subjects  Search modes - Boolean/Phrase |

*Note*. DE = indexing term. AB = terms in the abstract. “ “ = phrase search. * = truncation. #/? = wild card.

**Supplementary Table S2.** Search strategy employed in the *APA PsychInfo* database using the *EBSCOhost* platform.

| **Search ID** | **Search Terms** | **Search Options** |
| --- | --- | --- |
| S1 | (DE "Schizophrenia") OR (DE "Schizophreniform Disorder") OR (DE "Schizotypal Personality Disorder") OR (DE "Brief Psychotic Disorder") OR (DE "Psychosis") OR (DE "Schizoaffective Disorder") OR (DE "Delusional Disorder") | Expanders - Apply equivalent subjects  Search modes - Boolean/Phrase |
| S2 | AB schizo* or "schizophrenia spectrum disorder*" or "non-affective psychos*" or "schizotypal personality disorder" or "psychotic disorder*" or "schizophreniform disorder" or "schizoaffective disorder" or "brief psychotic disorder" or psychos?s or "delusional disorder" | Expanders - Apply equivalent subjects  Search modes - Boolean/Phrase |
| S3 | S1 OR S2 | Expanders - Apply equivalent subjects  Search modes - Boolean/Phrase |
| S4 | DE "Visual Perception" | Expanders - Apply equivalent subjects  Search modes - Boolean/Phrase |
| S5 | AB "early visual processing" or "basic visual processing" or "visual process*" or "visual percept*" or "visual function*" or "visual abnormalit*" or "visuo-perceptual deficit*" or "visual information" | Expanders - Apply equivalent subjects  Search modes - Boolean/Phrase |
| S6 | S4 OR S5 | Expanders - Apply equivalent subjects  Search modes - Boolean/Phrase |
| S7 | (DE "Social Cognition") OR (DE "Mentalization") OR (DE "Facial Affect Recognition") OR "DE "Social Perception") OR (DE "Attribution") OR (DE "Social Adjustment") OR (DE "Theory of Mind") OR (DE "Empathy") OR (DE "Emotion Recognition") | Expanders - Apply equivalent subjects  Search modes - Boolean/Phrase |
| S8 | AB "social cogniti*" or "social percept*" or "social knowledge" or "social judg#ment" or "social cue*" or "theory of mind" or "ToM" or mentali* or "mind reading" or empathy or "emotion recognition" or "affect recognition" or "emotion perception*" or "affect perception" or attribution or "attributional bias" | Expanders - Apply equivalent subjects  Search modes - Boolean/Phrase |
| S9 | S7 OR S8 | Expanders - Apply equivalent subjects  Search modes - Boolean/Phrase |
| S10 | (DE "Psychosocial Outcomes") OR (DE "Social Functioning") OR (DE "Social Behavior") OR (DE "Social Interaction") OR (DE "Social Skills") OR (DE "Quality of Life") OR (DE "Life Satisfaction") OR (DE "Social Adjustment") OR (DE "Life Skills") | Expanders - Apply equivalent subjects  Search modes - Boolean/Phrase |
| S11 | AB "functional outcome" or "social outcome" or "social functioning" or "social-cognitive performance" or "work functioning" or "occupational functioning" or "daily functioning" or "vocational functioning" or "quality of life" or "life satisfaction" or "social relationships" or "social integration" or "social behavio#r" or "social adjustment" or "social dysfunction" or "independent living skills" or "skills of daily living" or "life skills" or "social skill*" or "social interaction" or "social participation" or "psychosocial outcome*" or "social adapt*" or "social disabilit*" or "social competenc*" | Expanders - Apply equivalent subjects  Search modes - Boolean/Phrase |
| S12 | S10 OR S11 | Expanders - Apply equivalent subjects  Search modes - Boolean/Phrase |
| S13 | S3 AND S6 AND S9 AND S12 | Limiters - Publication Date: 19770101-20231131; Peer Reviewed; Publication Type: Peer Reviewed Journal; English language; Document Type: Journal Article  Expanders - Apply equivalent subjects  Search modes - Boolean/Phrase |
| S14 | S3 AND S6 AND S12 | Limiters - Publication Date: 19770101-20231131; Peer Reviewed; Publication Type: Peer Reviewed Journal; English language; Document Type: Journal Article  Expanders - Apply equivalent subjects  Search modes - Boolean/Phrase |

*Note.* DE = indexing term (APA Thesaurus). AB = terms in the abstract. “ “ = phrase search. * = truncation. #/? = wild card.

**Supplementary Table S3.** Search strategy employed in the *MEDLINE Complete* database using the *EBSCOhost* platform.

| **Search ID** | **Search Terms** | **Search Options** |
| --- | --- | --- |
| S1 | (MH "Schizophrenia+") OR (MH "Schizophrenia Spectrum and Other Psychotic Disorders") OR (MH "Schizotypal Personality Disorder") OR (MH "Psychotic Disorders") OR (MH "Schizophrenia, Paranoid") | Expanders - Apply equivalent subjects  Search modes - Boolean/Phrase |
| S2 | AB schizo* or "schizophrenia spectrum disorder*" or "non-affective psychos*" or "schizotypal personality disorder" or "psychotic disorder*" or "schizophreniform disorder" or "schizoaffective disorder" or "brief psychotic disorder" or psychos?s or "delusional disorder" | Expanders - Apply equivalent subjects  Search modes - Boolean/Phrase |
| S3 | S1 OR S2 | Expanders - Apply equivalent subjects  Search modes - Boolean/Phrase |
| S4 | (MH "Visual Perception+") | Expanders - Apply equivalent subjects  Search modes - Boolean/Phrase |
| S5 | AB "early visual processing" or "basic visual processing" or "visual process*" or "visual percept*" or "visual function*" or "visual abnormalit*" or "visuo-perceptual deficit*" or "visual information" | Expanders - Apply equivalent subjects  Search modes - Boolean/Phrase |
| S6 | S4 OR S5 | Expanders - Apply equivalent subjects  Search modes - Boolean/Phrase |
| S7 | (MH "Social Cognition") OR (MH "Social Perception") OR (MH "Theory of Mind") OR (MH "Mentalization") OR (MH "Empathy") | Expanders - Apply equivalent subjects  Search modes - Boolean/Phrase |
| S8 | AB "social cogniti*" or "social percept*" or "social knowledge" or "social judg#ment" or "social cue*" or "theory of mind" or "ToM" or mentali* or "mind reading" or empathy or "emotion recognition" or "affect recognition" or "emotion perception*" or "affect perception" or attribution or "attributional bias" | Expanders - Apply equivalent subjects  Search modes - Boolean/Phrase |
| S9 | S7 OR S8 | Expanders - Apply equivalent subjects  Search modes - Boolean/Phrase |
| S10 | (MH "Social Interaction") OR (MH "Social Skills") OR (MH "Psychosocial Functioning") OR (MH "Quality of Life") OR (MH "Social Behavior") OR (MH "Social Adjustment") OR (MH "Social Participation") | Expanders - Apply equivalent subjects  Search modes - Boolean/Phrase |
| S11 | AB "functional outcome" or "social outcome" or "social functioning" or "social-cognitive performance" or "work functioning" or "occupational functioning" or "daily functioning" or "vocational functioning" or "quality of life" or "life satisfaction" or "social relationships" or "social integration" or "social behavio#r" or "social adjustment" or "social dysfunction" or "independent living skills" or "skills of daily living" or "life skills" or "social skill*" or "social interaction" or "social participation" or "psychosocial outcome*" or "social adapt*" or "social disabilit*" or "social competenc*" | Expanders - Apply equivalent subjects  Search modes - Boolean/Phrase |
| S12 | S10 OR S11 | Expanders - Apply equivalent subjects  Search modes - Boolean/Phrase |
| S13 | S3 AND S6 AND S9 AND S12 | Limiters - Publication Date: 19770101-20231131; English Language; Publication Type: Journal Article; Peer Reviewed  Expanders - Apply equivalent subjects  Search modes - Boolean/Phrase |
| S14 | S3 AND S6 AND S12 | Limiters - Publication Date: 19770101-20231131; English Language; Publication Type: Journal Article; Peer Reviewed  Expanders - Apply equivalent subjects  Search modes - Boolean/Phrase |

*Note.* MH = indexing term (MesH). AB = terms in the abstract. “ “ = phrase search. * = truncation. #/? = wild card.

**Supplementary Table S4.** Search strategy employed in the *Embase* database using the *Ovid* platform.

| **Search ID** | **Search Terms** |
| --- | --- |
| S1 | schizophrenia/ or psychosis/ or schizophrenia spectrum disorder/ or schizotypal personality disorder/ or schizophreniform disorder/ or schizoaffective psychosis/ or brief psychotic disorder/ or delusional disorder/ |
| S2 | (schizo* or "schizophrenia spectrum disorder*" or "non-affective psychos*" or "schizotypal personality disorder" or "psychotic disorder*" or "schizophreniform disorder" or "schizoaffective disorder" or "brief psychotic disorder" or psychos#s or "delusional disorder").ab. |
| S3 | S1 OR S2 |
| S4 | vision/ or color discrimination/ or visual acuity/ or visual information/ |
| S5 | ("early visual processing" or "basic visual processing" or "visual process*" or "visual percept*" or "visual function*" or "visual abnormalit*" or "visuo-perceptual deficit*" or "visual information").ab. |
| S6 | S4 OR S5 |
| S7 | exp social cognition/ |
| S8 | ("social cogniti*" or "social percept*" or "social knowledge" or "social judg?ment" or "social cue*" or "theory of mind" or "ToM" or mentali* or "mind reading" or empathy or "emotion recognition" or "affect recognition" or "emotion perception*" or "affect perception" or attribution or "attributional bias").ab. |
| S9 | S7 OR S8 |
| S10 | social interaction/ or social behavior/ or "quality of life"/ or life satisfaction/ or social adaptation/ or social disability/ or social competence/ |
| S11 | ("functional outcome" or "social outcome" or "social functioning" or "social-cognitive performance" or "work functioning" or "occupational functioning" or "daily functioning" or "vocational functioning" or "quality of life" or "life satisfaction" or "social relationships" or "social integration" or "social behavio?r" or "social adjustment" or "social dysfunction" or "social disability" or "independent living skills" or "skills of daily living" or "life skills" or "social skill*" or "social interaction" or "social participation" or "psychosocial outcome*" or "social adaptation" or "social competence").ab. |
| S12 | S10 OR S11 |
| S13 | S3 AND S6 AND S9 AND S12 |
| S14 | limit 13 to (english language and embase and yr="1977 -Current" and article and journal) |
| S15 | S3 AND S6 AND S12 |
| S16 | limit 15 to (english language and embase and yr="1977 -Current" and article and journal) |

*Note.* AB = terms in the abstract. “ “ = phrase search. * = truncation. #/? = wild card.

**Supplementary Table S5.** Search strategy employed in the *Web of Science* database using the *Clarivate* platform.

| **Search ID** | **Search Terms** |
| --- | --- |
| S1 | TS=(schizo* or "schizophrenia spectrum disorder*" or "non-affective psychos*" or "schizotypal personality disorder" or "psychotic disorder*" or "schizophreniform disorder" or "schizoaffective disorder" or "brief psychotic disorder" or psychos?s or "delusional disorder" |
| S2 | TS=("early visual processing" or "basic visual processing" or "visual process*" or "visual percept*" or "visual function*" or "visual abnormalit*" or "visuo-perceptual deficit*" or "visual information") |
| S3 | TS=("social cogniti*" or "social percept*" or "social knowledge" or "social judg$ment" or "social cue*" or "theory of mind" or "ToM" or mentali* or "mind reading" or empathy or "emotion recognition" or "affect recognition" or "emotion perception*" or "affect perception" or attribution or "attributional bias") |
| S4 | TS=("functional outcome" or "social outcome" or "social functioning" or "social-cognitive performance" or "work functioning" or "occupational functioning" or "daily functioning" or "vocational functioning" or "quality of life" or "life satisfaction" or "social relationships" or "social integration" or "social behavio$r" or "social adjustment" or "social dysfunction" or "independent living skills" or "skills of daily living" or "life skills" or "social skill*" or "social interaction" or "social participation" or "psychosocial outcome*" or "social adapt*" or "social disabilit*" or "social competenc*") |
| S5 | S1 AND S2 AND S3 AND S4 |
| S6 | #5 and Article (Document Types) and English (Languages) and Timespan: 1977-01-01 to 2023-11-10 (Publication Date) |
| S7 | S1 AND S2 AND S4 |
| S8 | #7 and Article (Document Types) and English (Languages) and Timespan: 1977-01-01 to 2023-11-10 (Publication Date) |

*Note.* TS = Topic search. “ “ = phrase search. * = truncation. $/? = wild card.

**Supplementary Table S6.** Moderator Analyses for the Relationship Between Early Visual Processing and Functional Outcomes in Schizophrenia Spectrum Disorders.

| Moderator Variable | k | Coefficient (ß) | Std. Error | 95% CI | p-value | I² (%) | tau² | Q_res (df) | p (Q_res) |
| --- | --- | --- | --- | --- | --- | --- | --- | --- | --- |
| Age | 8 | -0.001 | 0.012 | [-0.024, 0.034] | 0.943 | 0.00 | 3.2×10⁻⁸ | 7.26 (6) | 0.298 |
| Gender | 8 | 0.001 | 0.003 | [-0.005, 0.007] | 0.831 | 0.00 | 3.3×10⁻⁷ | 7.22 (6) | 0.301 |
| Education | 5 | 0.007 | 0.097 | [-0.182, 0.197] | 0.939 | 0.00 | 2.8×10⁻⁷ | 0.44 (3) | 0.932 |
| Illness Duration | 7 | 0.000 | 0.013 | [-0.025, 0.025] | 0.980 | 0.00 | 8.6×10⁻⁸ | 6.54 (5) | 0.258 |
| Antipsychotic Treatment | 6 | 0.009 | 0.012 | [-0.014, 0.032] | 0.444 | 0.00 | 5.3×10⁻⁷ | 3.05 (4) | 0.550 |
| Daily CPZ Equivalents | 3 | 0.000 | 0.000 | [-0.000, 0.001] | 0.351 | 48.21 | 0.020 | 1.93 (1) | 0.165 |
| Methodological Quality | 8 | 0.023 | 0.040 | [-0.054, 0.101] | 0.551 | 0.00 | 1.6×10⁻⁷ | 6.90 (6) | 0.330 |

*Note.* The moderator “Ethnicity” was not included in the meta-regression due to insufficient observations. k = Number of studies (observations) included in each meta‐regression; I² = Proportion of variance due to between‐study heterogeneity; tau² = Estimated variance in true effect sizes; Q_res = Residual heterogeneity after accounting for the moderator; p (Q_res) = Significance test for the remaining heterogeneity; CPZ = Chlorpromazine.

**Supplementary Table S7.** Moderator Analyses for the Relationship Between Early Visual Processing and Social Cognition in Schizophrenia Spectrum Disorders.

| Moderator Variable | k | Coefficient (ß) | Std. Error | 95% CI | p-value | I² (%) | tau² | Q_res (df) | p (Q_res) |
| --- | --- | --- | --- | --- | --- | --- | --- | --- | --- |
| Age | 5 | 0.000 | 0.028 | [-0.054, 0.055] | 0.991 | 0.01 | 5.4×10⁻⁷ | 2.20 (3) | 0.531 |
| Gender | 5 | 0.003 | 0.004 | [-0.004, 0.010] | 0.463 | 0.01 | 8.9×10⁻⁷ | 1.67 (3) | 0.645 |
| Education | 4 | 0.024 | 0.097 | [-0.166, 0.214] | 0.802 | 0.01 | 4.3×10⁻⁷ | 1.55 (2) | 0.461 |
| Illness Duration | 5 | -0.003 | 0.019 | [-0.040, 0.034] | 0.870 | 0.00 | 8.7×10⁻⁸ | 2.18 (3) | 0.537 |
| Antipsychotic Treatment | 4 | -0.006 | 0.013 | [-0.033, 0.020] | 0.640 | 0.00 | 2.5×10⁻⁷ | 1.39 (2) | 0.498 |
| Methodological Quality | 5 | -0.042 | 0.042 | [-0.126, 0.042] | 0.324 | 0.00 | 1.3×10⁻⁷ | 1.23 (3) | 0.746 |

*Note.* The moderators “Ethnicity” and *“*Chlorpromazine Daily Equivalent” were not included in the meta-regression due to insufficient observations. k = Number of studies (observations) included in each meta‐regression; I² = Proportion of variance due to between‐study heterogeneity; tau² = Estimated variance in true effect sizes; Q_res = Residual heterogeneity after accounting for the moderator; p (Q_res) = Significance test for the remaining heterogeneity.

**Supplementary Table S8.** Moderator Analyses for the Relationship Between Social Cognition and Functional Outcomes in Schizophrenia Spectrum Disorders.

| Moderator Variable | k | Coefficient (ß) | Std. Error | 95% CI | p-value | I² (%) | tau² | Q_res (df) | p (Q_res) |
| --- | --- | --- | --- | --- | --- | --- | --- | --- | --- |
| Age | 4 | 0.008 | 0.028 | [-0.047, 0.063] | 0.785 | 0.00 | 2.1×10⁻⁸ | 0.64 (2) | 0.725 |
| Gender | 4 | -0.002 | 0.004 | [-0.010, 0.005] | 0.543 | 0.00 | 1.1×10⁻⁷ | 0.35 (2) | 0.840 |
| Education | 4 | 0.006 | 0.097 | [-0.184, 0.196] | 0.949 | 0.00 | 6.5×10⁻⁸ | 0.71 (2) | 0.700 |
| Illness Duration | 4 | 0.009 | 0.023 | [-0.036, 0.055] | 0.685 | 0.01 | 5.8×10⁻⁷ | 0.55 (2) | 0.758 |
| Antipsychotic Treatment | 4 | -0.006 | 0.013 | [-0.033, 0.020] | 0.631 | 0.00 | 1.2×10⁻⁷ | 0.49 (2) | 0.784 |
| Methodological Quality | 4 | 0.019 | 0.044 | [-0.068, 0.105] | 0.673 | 0.00 | 1.5×10⁻⁷ | 0.54 (2) | 0.764 |

*Note.* The moderators “Ethnicity” and *“*Chlorpromazine Daily Equivalent” were not included in the meta-regression due to insufficient observations. k = Number of studies (observations) included in each meta‐regression; I² = Proportion of variance due to between‐study heterogeneity; tau² = Estimated variance in true effect sizes; Q_res = Residual heterogeneity after accounting for the moderator; p (Q_res) = Significance test for the remaining heterogeneity.
